# The burden of suicide in the Emergency Room: a 3-year single center experience

**DOI:** 10.1192/j.eurpsy.2025.726

**Published:** 2025-08-26

**Authors:** C. Mauceri, A. Prebilic, L. Isabella, F. Gavelli, E. Gambaro, C. Gramaglia, P. Zeppegno

**Affiliations:** 1 Department of Traslational Medicine, Università degli Studi del Piemonte Orientale; 2 Emergency Medicine Unit; 3 S.C. Psichiatria, AOU “Maggiore della Carità”, Novara, Italy

## Abstract

**Introduction:**

Suicide is one of the leading causes of death worldwide with more than 700.000 deaths per year. Its risk factors encompass socioeconomic elements, psychological burdens and mental health disorders, and biological variables. Still, the understanding of different risk factors and their interplay remains incomplete. Suicide attempts are estimated to be from ten to forty times more frequent than suicide deaths.

**Objectives:**

The main objective of this ongoing research is to investigate socio-demographic and clinical variables of suicidal patients in an emergency setting. The secondary objective is to analyze risk factors, clinical presentation and outcome comparing patients with single or repeated psychiatric assessments in the emergency room.

**Methods:**

This study retrospectively analyzes data from patients who underwent psychiatric evaluation in the ER setting following suicide ideation and suicide behavior, at the “Maggiore della Carità” hospital in Novara, from January 2021 to January 2023. Initial descriptive statistics have been performed using frequencies and percentages. Additional statistical analyses on collected data are in development.

**Results:**

Preliminary results showed that 1674 patients were admitted at the ER for psychiatric consultation in the three-year period, 177 of whom evaluated for SI and SB (females = 116, 66.3%) mainly from Italy (n = 151, 88.3%). The mean age was 41.26 ± 18.54 years (range = 16-88 years). The majority of patients were unmarried (=79, 53.0%), employed (=40, 34.5%), with lower secondary education (=36, 38.3%) and living with family (=108, 71.5%). A pre-existing psychiatric condition was reported nearly in two-thirds of individuals (=129, 74.1%); linked predominantly with personality disorders (=47, 34.6%), major depressive disorders (=30, 22.1%) and anxiety disorders (=13, 9.6%). The most common methods for suicidal attempts were overdose with medication (=106, 60.6%) and self-inflicted injuries (=31, 17.7%). A single psychiatric assessment was conducted nearly in the totality of cases (=147, 86.4%), while a minority of records consisted of repeated access from the same patient (=30, 13.6%).

**Conclusions:**

Initial findings are in line with current literature on suicide risk factors. Further statistical analyses will be conducted to search for known associations between risk factors, and to shed light on the most important elements to consider when evaluating suicide ideation and suicide behavior risks in an emergency setting.

**Disclosure of Interest:**

None Declared

